# Systemic inflammation indices generated from first-trimester blood tests can be potential biomarkers for predicting preeclampsia

**DOI:** 10.1038/s41371-026-01169-y

**Published:** 2026-06-10

**Authors:** Yu Chen, Jingyang Li, Xiaoshuang Li, Yihui Pan, Ying Li, Ying Gu, Qi Chen

**Affiliations:** 1 Department of Obstetrics, Wuxi Maternity and Child Health Care Hospital, Affiliated to Jiangnan University, Wuxi, China; 2 Department of Obstetrics and Gynaecology, Faculty of Medical and Health Sciences, The University of Auckland, Auckland, New Zealand

**Keywords:** Diagnosis, Medical research

## Abstract

Preeclampsia is a leading cause of maternal and neonatal morbidity and mortality, affecting 3 to 8% of pregnancies. Currently, no clinically useful predictive biomarker exists. Preeclampsia is characterised by systemic inflammatory responses that may be triggered by placental materials released into the maternal circulation, leading to an overly active maternal immune system. Recently, systemic inflammation indices have been used as indicators of inflammation in various diseases. Here, we investigated whether systemic inflammation indices derived from peripheral blood tests in the first trimester could serve as potential predictors of preeclampsia. Blood samples, ranging from 6 to 12 weeks, were collected from 109 women who later developed preeclampsia and 177 women who did not develop any complications. The counts of white blood cells, neutrophils, lymphocytes, monocytes, eosinophils, basophils, and platelets were obtained, and systemic inflammation indices, including the systemic immune inflammation index (SII), the systemic inflammation response index (SIRI), the neutrophil-to-lymphocyte ratio (NLR), and the pan-immune inflammation value (PIV), were calculated. Significantly higher counts of white blood cells, neutrophils, lymphocytes, and monocytes, but not eosinophils and basophils, were observed in women who later developed preeclampsia (*p* < 0.001). Additionally, all four indices were significantly elevated in women who later developed preeclampsia (*p* < 0.001). The increased indices were primarily associated with late-onset preeclampsia. No significant differences in these indices were observed between women who developed preeclampsia with or without severe features. Our study demonstrates that systemic inflammation indices derived from first-trimester blood tests may serve as additional predictors for preeclampsia when combined with other reported biomarkers.

## Introduction

Preeclampsia is a leading cause of maternal and neonatal morbidity and mortality worldwide, affecting 3 to 8% of all pregnancies [1]. Although the pathogenesis is not fully understood, placental hypoxia, placental inflammation, oxidative stress and metabolic dysfunctions are associated with the development of preeclampsia [2–4]. Despite its clinical significance, no reliable predictive biomarkers exist for early detection. The ratio of soluble fms-like tyrosine kinase 1 (sFlt-1) to placental growth factor (PlGF) is used as a potential predictive biomarker [5]. Preeclampsia is characterised by a systemic inflammatory response that may be potentially triggered by placental materials, such as placental extracellular vesicles (EVs) released into the maternal circulation, leading to excessive maternal immune activation [6].

Recently, systemic inflammation indices have been explored as early biomarkers in various diseases and clinical conditions, including pregnancy complications [7], as early biomarkers for inflammation-associated diseases [8, 9]. These systemic inflammation indices include the systemic immune inflammation index (SII), the systemic inflammation response index (SIRI), the neutrophil-to-lymphocyte ratio (NLR), and the pan-immune inflammation value (PIV). While lower counts of platelets are commonly observed in preeclampsia at the time of diagnosis (review in [10]), in the current literature, studies on systemic immune inflammation indices as predictive markers for preeclampsia remain inconclusive, potentially due to variations in sample size and sampling time [11–14]. Some studies indicated that a lower SII in the first trimester correlated with an increased risk of developing preeclampsia, but those studies were limited by their small sample sizes and incomplete data [15, 16]. Conversely, another study reported that increased SIRI and PIV, but not SII and NLR, measured in the first trimester, may serve as potential predictive markers for preeclampsia [17].

Preeclampsia is clinically classified into early-onset and late-onset forms with distinct underlying mechanisms. It has been known that the early-onset form is primarily associated with placental dysfunction resulting from defective placentation. On the other hand, the late-onset form is more associated with maternal factors, such as maternal metabolic dysfunction, suggesting relatively normal placental development. Additionally, preeclampsia is also categorized into preeclampsia with or without severe features (formerly severe or mild preeclampsia) [18]. Preeclampsia with severe features is more strongly related to placental dysfunction, while preeclampsia without severe features is often driven by maternal factors (reviewed in [1]).

Systemic immune inflammation indices are currently being investigated for predicting preeclampsia, as they can be obtained from a routine, non-invasive, and cost-effective blood test. However, no study has comprehensively evaluated their role in predicting preeclampsia in the first trimester while accounting for disease severity. Therefore, in this retrospective study with a relatively large sample size, we investigated whether systemic immune inflammation indices generated from peripheral blood tests in the first trimester could serve as potential predictive biomarkers for predicting preeclampsia.

## Methods and materials

This retrospective study received approval from the Ethical Committee of Wuxi Maternity and Child Health Care Hospital, Jiangnan University, Wuxi, China (approval numbers:2024-06-1024-54). The Ethical Committee waived the patient’s consent form due to the data collected from the hospital database. This study was performed in accordance with the Medical Association Declaration of Helsinki.

### Study cohort

In this retrospective study, women diagnosed with preeclampsia were identified in our hospital databases from January 2022 to December 2023. After excluding those who developed chronic hypertension (before 20 weeks of gestation), or had a history of chronic hypertension, or had a history of preeclampsia, or were conceived by in vitro fertilisation (IVF), a total of 109 pregnant women were included. Additionally, 177 pregnant women who did not develop any maternal or neonatal complications until delivery were included as controls. Blood samples from the first trimester (6–12 weeks) of these women were collected. The mean (± SD) gestation age at sampling was 8.7 ± 2.3 or 8.6 ± 2.4 for cases or controls, respectively. The counts of white blood cells, neutrophils, lymphocytes, monocytes, eosinophils, basophils, and platelets were obtained, along with systemic inflammation indices. Of 109 women who developed preeclampsia later, 17 (16%) women developed early-onset preeclampsia, and 26 (24%) developed preeclampsia with severe features. Of the 17 women who developed early-onset preeclampsia, 5 (29%) developed preeclampsia with severe features. Of the 92 women who developed late-onset preeclampsia, 21 (23%) developed preeclampsia with severe features.

Data on maternal age, parity, gravidity, gestational age of onset, gestational age at delivery, systolic and diastolic blood pressure at the time of onset, birthweight, and maternal body weight (both pre-pregnancy and before delivery), and height for calculation of maternal body mass index (BMI) were collected from the hospital databases. BMI was classified according to the WHO classification for Asian/Indian women. The BMIs for underweight, normal weight, overweight, and obese are under 18.4 kg/m^2^, 18.5–22.99 kg/m^2^, 23–27.49 kg/m^2^, and over 27.50 kg/m^2^, respectively [19]. Mean weekly BMI change was calculated by dividing the difference in BMI before pregnancy and before delivery by the gestational age in weeks.

Preeclampsia was defined as when maternal systolic blood pressure was ≥ 140 mmHg and/or diastolic blood pressure was ≥ 90 mmHg on two occasions, between 6 h with or without proteinuria (>300 mg in 24 h), and/or impaired liver function, and/or lower platelet count, after 20 weeks of gestation, followed by international guidelines [20]. Systolic blood pressure ≥ 160 mmHg and/or diastolic blood pressure ≥ 110 mmHg, with impaired liver or renal function and/or a lower platelet count, was defined as preeclampsia with severe features. The gestational week of onset, before or after 34 weeks, was defined as early or late-onset preeclampsia.

The systemic immune-inflammation index (SII) was calculated as platelet count × neutrophil /lymphocyte count (10^9/L). The systemic inflammation response index (SIRI) was calculated by neutrophil count × monocyte count/lymphocyte count (10^9/L). The neutrophil-to-lymphocyte ratio (NLR) was calculated by neutrophil /lymphocyte count (10^9/L). Pan-immune inflammation value (PIV) was calculated by neutrophil count × monocyte count × platelet count/lymphocyte count (10^9/L). The cut-off value of each index was calculated by identifying the point on the curve with a minimum distance from the upper-left corner of the unit square, maximizing Youden’s index [21].

### Statistical analysis

Data for blood tests and indices were presented as medians and interquartile ranges (IQRs). Data for clinical characteristics were presented as mean ± standard deviation (SD) or as percentages. T-test (Mann-Whitney test) and Chi-square test were performed using Prism GraphPad (version 10). Receiver operating characteristic (ROC) curves and the area under the ROC curve (AUC) were generated for statistical analysis using IBM SPSS (version 30.0). Statistical difference was considered when *p* < 0.05.

## Results

The clinical characteristics are presented in Table 1. The maternal age in women who developed preeclampsia later was significantly older than that of controls (32.6 vs 30.3 years, *p* < 0.001). The delivery weeks in women who developed preeclampsia later were also significantly earlier than controls (37^+3^ vs 39^+3^ weeks, *p* < 0.001). Additionally, the BMI before pregnancy was significantly higher in women who developed preeclampsia later compared to controls (25.0 vs 21.5 kg/m^2^, *p* < 0.001).

**Table 1:** Clinical characteristics of study participants.

	Preeclampsia (*n* = 109)	Controls (*n* = 177)	*P* value
Maternal age (years, mean/SD)	32.6 ± 4.7	30.3 ± 3.9	*p* < 0.001
Gravidity (n, %))			
1	56 ((51%)	75 (43%)	*p* = 0.248
2	25 (23%)	55 (31%)	
≥ 3	28 (26%)	46 (26%)	
Parity (n, %)			
0	79 (72%)	117 (66%)	*p* = 0.097
1	24 (22%)	56 (32%)	
≥2	6 (6%)	4 (2%)	
Onset weeks (mean/SD)	36^+2.7^ ± 3.5^+2.0^	N/A	N/A
Systolic blood pressure (mmHg, mean/SD)	152 ± 13	N/A	N/A
Diastolic blood pressure (mmHg, mean/SD)	94 ± 9	N/A	N/A
Delivery weeks (mean/SD)	37^+3^ ± 2.5^+1.9^	39^+3^ ± ^1+2.0^	*p* < 0.001
Delivery mode			
Cesarean section (n, %)	92 (84%)	45 (25%)	*p* < 0.001
Vaginal delivery (n, %)	17 (16%)	132 (75%)	
Birthweight (g, mean/SD)	3318 ± 391	2738 ± 779	*p* < 0.001
BMI pre-pregnancy (Kg/m^2^, mean/SD)	25.07 ± 4.2	21.49 ± 2.7	*p* < 0.001

*N/A* not applicable.

We first compared the counts of white cells, neutrophils, lymphocytes, monocytes, eosinophils, basophils, and platelets between the two groups (Table 2). Significantly higher counts of white cells, neutrophils, lymphocytes, monocytes, and platelets, but not eosinophils or basophils, were observed in the first trimester in women who later developed preeclampsia compared with controls. The systemic inflammation indices, including SII, SIRI, NLR, and PIV, were significantly higher in women who later developed preeclampsia than in the controls (Table 2).

**Table 2:** Complete blood cell counts and systemic inflammation indices measured in the first trimester in the study cohort.

	Preeclampsia (*n* = 109)	Controls (*n* = 117)	*P* value
Whole white cell counts (10^9/L, median/IQR)	9.1 (7.67, 10.83)	7.48 (6.42, 9.28)	***p*** < **0.001**
Neutrophil (10^9/L, median/IQR)	6.6 (5.4, 8.1)	5.4 (4.3, 6.75)	***p*** < **0.001**
Monocyte (10^9/L, median/IQR)	0.46 (0.38, 0.6)	0.4 (0.33, 0.5)	***p*** < **0.001**
Lymphocyte (10^9/L, median/IQR)	1.9 (1.55, 2.2)	1.7 (1.4, 2.0)	***p*** = **0.006**
Platelet (10^9/L, median/IQR)	249 (217, 293)	230 (198, 266)	***p*** = **0.003**
Eosinophils (10^9/L, median/IQR)	0.07 (0.00, 0.12)	0.05 (0.00, 0.10)	*P* = 0.052
Basophils (10^9/L, median/IQR)	0.02 (0.00, 0.03)	0.02 (0.00, 0.03)	*P* = 0.881
SII (median/IQR)	940 (688, 1157)	708 (514, 949)	***p*** < **0.001**
SIRI (median/IQR)	1.81 (1.24, 2.22)	1.225 (0.87, 1.78)	***p*** < **0.001**
NLR (median/IQR)	3.62 (2.84, 4.23)	3.118 (2.37, 3.81)	***p*** = **0.002**
PIV (median/IQR)	432 (284, 635)	275 (180, 467)	***p*** < **0.001**

Blood sampling was performed at a mean gestational age of 8.7 weeks (SD ± 2.3) for cases and 8.6 weeks (SD ± 2.4) for controls. Data are expressed as median (IQR).

Preeclampsia is clinically categorized into early-onset and late-onset, as well as preeclampsia with or without severe features. We then compared the systemic inflammation indices between different forms of preeclampsia and controls. As shown in Table 3, there were no differences in SII, SIRI, NLR, and PIV between early-onset preeclampsia and the controls. In contrast, SIRI, SII, NLR, and PIV were significantly increased in late-onset preeclampsia compared to controls. Additional analysis showed no statistical differences in SII, SIRI, NLR, or PIV between early-onset and late-onset preeclampsia (Table 3).

**Table 3:** The comparison of systemic inflammation indices in women who developed preeclampsia later, according to the onset time.

	Early-onset preeclampsia (*n* = 17)	Controls (*n* = 117)	*P* value
SII (median/IQR)	719 (554, 1039)	708 (514, 949)	*p* = 0.593
SIRI (median/IQR)	1.288 (0.91, 1.96)	1.225 (0.87, 1.78)	*p* = 0.556
NLR (median/IQR)	3.267 (2.28, 4.08)	3.118 (2.37, 3.81)	*p* = 0.953
PIV (median/IQR)	304 (197, 508)	275 (180, 467)	*p* = 0.458
	Late-onset preeclampsia (*n* = 92)	Controls (*n* = 117)	*P* value
SII (median/IQR)	942 (719, 1223)	708 (514, 949)	***p*** < **0.001**
SIRI (median/IQR)	1.88 (1.28, 2.29)	1.225 (0.87, 1.78)	***p*** < **0.001**
NLR (median/IQR)	3.67 (2.88, 4.27)	3.118 (2.37, 3.81)	***p*** < **0.001**
PIV (median/IQR)	438 (299. 671)	275 (180, 467)	***p*** < **0.001**
	Early-onset preeclampsia (*n* = 17)	Late-onset preeclampsia (*n* = 92)	*P* value
SII (median/IQR)	719 (554, 1039)	942 (719, 1223)	*P* = 0.071
SIRI (median/IQR)	1.288 (0.91, 1.96)	1.88 (1.28, 2.29)	*P* = 0.050
NLR (median/IQR)	3.267 (2.28, 4.08)	3.67 (2.88, 4.27)	*P* = 0.109
PIV (median/IQR)	304 (197, 508)	438 (299. 671)	*P* = 0.060

Blood sampling was performed at a mean gestational age of 8.5 weeks (SD ± 2.8) for early onset, 8.7 weeks (SD ± 2.3) for late-onset preeclampsia, and 8.6 weeks (SD ± 2.4) for controls.

Additionally, significantly higher SII, SIRI, and PIV were observed in preeclampsia with severe features compared to the controls (Table 4). Significantly higher SII, SIRI, NLR, and PIV were observed in preeclampsia without severe features than in controls (Table 4).

**Table 4:** The comparison of systemic inflammation indices in women who developed preeclampsia later, according to the severity.

	Preeclampsia with severe features (*n* = 26)	Controls (*n* = 117)	*P* value
SII (median/IQR)	956 (646, 1226)	708 (514, 949)	***p*** = **0.012**
SIRI (median/IQR)	1.54 (1.23, 2.11)	1.225 (0.87, 1.78)	***p*** = **0.035**
NLR (median/IQR)	3.64 (2.84, 4.21)	3.118 (2.37, 3.81)	*p* = 0.081
PIV (median/IQR)	404 (264, 700)	275 (180, 467)	***p*** = **0.016**
	Preeclampsia without severe features (*n* = 83)	Controls (*n* = 117)	*P* value (Mann-Whitney)
SII (median/IQR)	905 (707, 1133)	708 (514, 949)	***p*** < **0.001**
SIRI (median/IQR)	1.87 (1.24, 2.26)	1.225 (0.87, 1.78)	***p*** < **0.001**
NLR (median/IQR)	3.62 (2.83, 4.26)	3.118 (2.37, 3.81)	***p*** = **0.004**
PIV (median/IQR)	440 (285, 630)	275 (180, 467)	***p*** < **0.001**

Blood sampling was performed at a mean gestational age of 8.8 weeks (SD ± 2.0), or 8.8 weeks (SD ± 2.6), or 8.6 weeks (SD ± 2.4) for preeclampsia with or without severe features, or for controls, respectively.

**Fig. 1:**
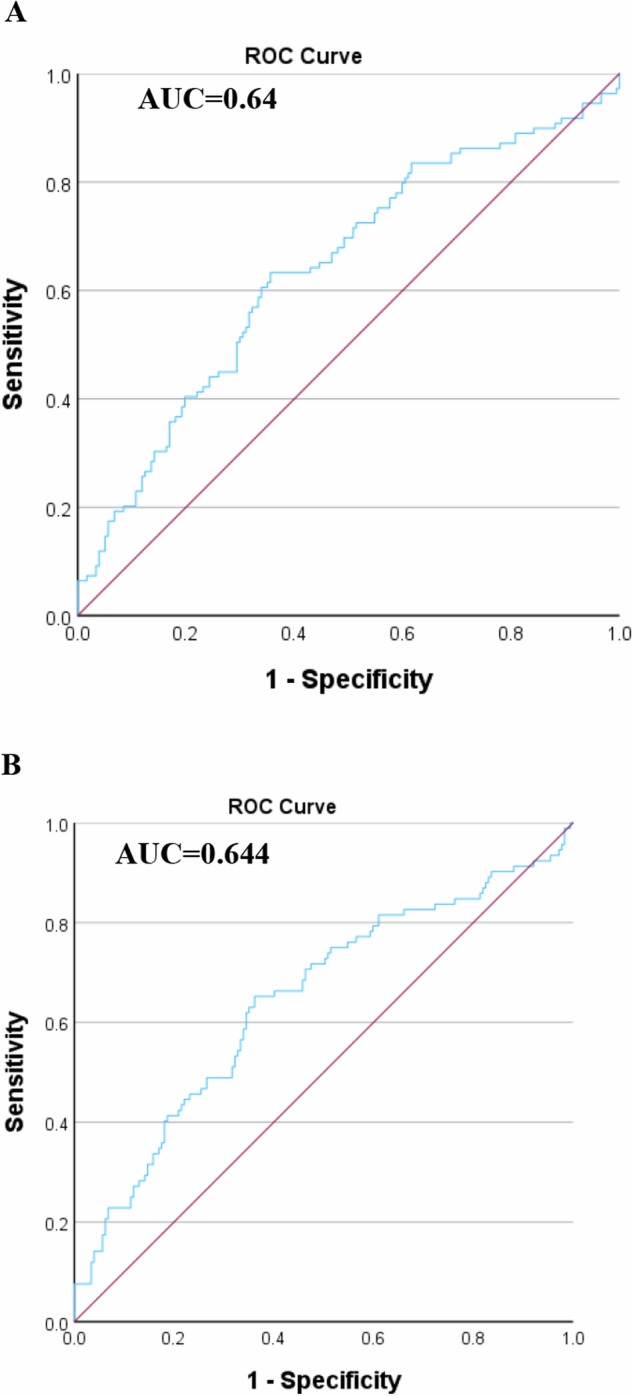
Predictive analysis using the area under the receiver operating characteristic (AUC). **A** The area under the receiver operating characteristic (AUC) was 0.640 for predicting preeclampsia using a combination of SIRI, SII, NLR, and PIV. **B** The area under the receiver operating characteristic (AUC) was 0.644 for predicting late-onset preeclampsia using a combination of SIRI, SII, NLR, and PIV.

We then calculated the predictive value using these four indices to predict preeclampsia and its subtype, late-onset preeclampsia. The optimal cut-off value, sensitivity, specificity, positive predictive value, and negative predictive value for each index are summarized in Table 5. Overall, the sensitivity of each index was over 60%, and its specificity was around 60%. When we combined these four indices together to predict preeclampsia, the AUC was 0.640 (95% CI: 0.573, 0.707; *p* < 0.001), with 63% sensitivity and 58% specificity (Fig. 1A). Furthermore, the AUC was 0.644 (95% CI: 0.573–0.716; *p* < 0.001), with 66% sensitivity and 58% specificity for predicting late-onset preeclampsia (Fig. 1B).

**Table 5:** The optimal cut-off value, sensitivity, specificity, PPV, and NPV for each index for predicting preeclampsia.

	Cut-off value	AUC (95% CI)	Sensitivity	Specificity	PPV (%)	NPV (%)	*P* value
SIRI	1.396	0.653 (0.588, 0.719)	64% (55–73%)	60% (51–69%)	59.8	64.1	***p*** < **0.001**
SII	799	0.639 (0.573, 0.705)	63% (54–72%)	58% (49–67%	58.3	62.7	***p*** < **0.001**
NLR	3.267	0.609 (0.542, 0.676)	62% (53–71%)	56% (47–65%)	56.8	61.3	***p*** = **0.002**
PIV	322	0.660 (0.595, 0.724)	67% (58–76%)	60% (51–69%)	60.9	66.3	***p*** < **0.001**

*PPV* positive predictive value, *NPV* negative predictive value.

To explore whether these indices correlate with obesity, we analyzed the BMI categories between the two groups. As shown in Supplementary Table 1, there was no statistical difference in BMI categories between the two groups, regardless of early-onset or preeclampsia with severe features (*p* = 0.777 and 0.468, respectively). However, the mean weekly BMI increase was significantly higher in late-onset preeclampsia than in early-onset preeclampsia (0.161 vs 0.115, *p* = 0.019).

## Discussion

In this retrospective study with a relatively large sample size, we found higher counts of white cells, neutrophils, lymphocytes, monocytes, and platelets in the first trimester among women who later developed preeclampsia than among those who did not. These increased blood cell counts consequently elevated systemic inflammation response indices (SII, SIRI, NLR, and PIV) in later women who developed preeclampsia. When these four indices were combined to predict the probability of preeclampsia, the resulting AUC was 0.640, with 63% sensitivity and 58% specificity. Increased systemic inflammation indices are more likely to be observed in women who develop late-onset preeclampsia.

Preeclampsia is characterized by a systemic inflammatory response that may be potentially triggered by pathological placental materials, such as extracellular vesicles (EVs). It has been well-reported that placental EVs derived from preeclamptic placentae triggered maternal endothelial cell dysfunctions [22, 23]. Studies have shown the interaction between placental EVs and maternal immune cells [24, 25], resulting in the modulation of immune response and producing inflammatory cytokines [26, 27]. Placental dysfunction occurs typically before the onset of clinical symptoms of preeclampsia, suggesting an early immune response in the initial stages of pregnancy. To date, there are no predictive biomarker(s) of preeclampsia for clinical use. Given that preeclampsia is an inflammatory disease, we investigated the potential predictive value of systemic inflammation response indices for predicting preeclampsia.

SII, SIRI, NLR, and PIV, calculated from peripheral neutrophil, monocyte, lymphocyte, and platelet counts, have recently been suggested as predictive markers for inflammation-associated pregnancy complications [12, 24, 25] and other gyanecological diseases such as endometriosis [26] and cesarean scar pregnancy [28]. Our current study found significantly higher indices of SII, SIRI, NLR, and PIV in the first trimester, as measured by blood tests, among women who later developed preeclampsia. Our findings are consistent with a recent study showing significantly higher SIRI and PIV in women who developed preeclampsia later [17]. Although that study [17] showed higher SII and NLR values in women who later developed preeclampsia, the difference did not reach statistical significance. In contrast, other studies showed a lower SII in preeclampsia. Unfortunately, those two studies did not mention the sampling time [15, 16].

The area under the ROC curve (AUC) is a widely used measure to assess the diagnostic or predictive accuracy of a test [29]. It represents the average sensitivity across all possible thresholds, with values above 0.5 indicating predictive capability or accuracy [30]. The AUC values below 0.80 are considered of limited clinical utility for diagnostic purposes. However, the acceptable AUC threshold depends on the disease context and its clinical implications. In a diagnostic study, a higher AUC is typically required for sufficient accuracy. Additionally, the 95% confidence interval provides important information on the precision and robustness of the model when interpreting AUC values. In our current study, combining four indices reached an AUC of 0.640 (95% CI: 0.573–0.707, *p* < 0.001), with 63% sensitivity and 58% specificity. These results indicate modest discriminative ability with moderate uncertainty. Preeclampsia is a multifactorial condition with multiple pathways involved. To date, no single biomarker or model has strong predictive power, partly because first-trimester biomarkers may capture only early biological signals rather than the full disease trajectory. The AUC of 0.640 reported in our current study is insufficient to serve as a single-screen tool for first-trimester risk prediction. Future longitudinal studies are required to confirm whether systemic inflammation indices can serve as predictive biomarkers. Combining with other factors, such as the sFlt-1/PIGF ratio and uterine artery Doppler, may provide additional value in predicting the development of preeclampsia. Currently, our model aims to stratify risk rather than provide a definitive diagnosis.

Preeclampsia is clinically categorized into early-onset and late-onset forms. It is well-known that early-onset preeclampsia is primarily associated with placental dysfunction, leading to insufficient uteroplacental blood flow, hypoxia, oxidative stress, and an anti-angiogenic state that disrupts maternal vascular health. While late-onset preeclampsia is more related to maternal factors, such as obesity. We then analyzed the systemic inflammation indices between women who later developed early-onset and late-onset preeclampsia. Significantly higher systemic inflammation indices were observed in women who later developed late-onset disease than in those who did not. However, this statistical significance was not observed in women who developed early-onset preeclampsia later. Further analysis showed no statistical differences in four systemic inflammation indices between women who later developed early-onset and late-onset preeclampsia. We acknowledge that the smaller sample size of the early-onset group (*n* = 17) may have limited statistical power. However, as shown in Table 3, the four indices showed higher values in the late-onset group, with a borderline significance (*p* = 0.05 for SIRI) and trend (*p* = 0.06 for PIV). This analysis further suggests that late-onset preeclampsia has a more pronounced inflammatory condition.

Preeclampsia is also clinically categorized into preeclampsia with and without severe features. But it remains unclear whether these two forms arise from distinct underlying mechanisms. While preeclampsia without severe features may be primarily driven by maternal factors, the exact pathophysiology remains poorly defined. A recent study [17] found no differences in these four systemic inflammation indices between preeclampsia with and without severe features. Consistent with these findings, we also found higher SII, SIRI, and PIV in women who later developed both forms of preeclampsia compared to those who did not. We also found no difference in these four indices between preeclampsia with and without severe features. These results suggest that both forms of preeclampsia may share common immune pathways, highlighting the role of maternal immune response in disease progression.

In conclusion, our study demonstrates elevated systemic inflammation indices generated from peripheral blood tests in the first trimester among women who later developed preeclampsia. Combining four indices reached an AUC of 0.640, with 63% sensitivity and 58% specificity. Peripheral blood tests are non-invasive and cost-effective. Our study suggests that these four indices, with modest predictive power, can serve as potential predictive biomarkers or risk-stratification factors for preeclampsia, as part of a combined screen model.

## Summary

### What is known about this topic

- Currently, no clinically useful predictive biomarker of preeclampsia exists.
- Systemic inflammation is associated with the development of preeclampsia.

### What this study adds

- Increased systemic inflammation indices generated from routine blood tests in the first trimester were observed in women who developed preeclampsia later.
- Systemic inflammation indices can potentially serve as a predictive biomarker of preeclampsia, with modest predictive power.

## Supplementary information

Supplementat Table

## Data Availability

The datasets used and analyzed during the current study are available from the corresponding author upon reasonable request.
