# Supplementary material for: Systemic inflammation indices generated from first-trimester blood tests can be potential biomarkers for predicting preeclampsia
Source: J Hum Hypertens. 2026 Jun 10;40(8):649–54. doi: 10.1038/s41371-026-01169-y (PMC13457073; doi:10.1038/s41371-026-01169-y)
Supplement: Supplementary file 1 [file 41371_2026_1169_MOESM1_ESM.doc]

**Supplementary Table**

**Table 1: The comparison of BMI categories between subtypes of preeclampsia**

|  | Early-onset (n=17) | Late-onset (n=92) | P value |
| --- | --- | --- | --- |
| Underweight (n, %) | 0 (0%) | 3 (3%) | p=0.777 |
| Normal weight (n, %) | 6 (35%) | 28 (30%) |  |
| Overweight (n, %) | 8 (47%) | 38 (41%) |  |
| Obese (n, %) | 3 (18%) | 23 (25%) |  |
|  | Preeclampsia with severe features (n=26) | Preeclampsia without severe features (n=83) | P value |
| Underweight (n, %) | 0 (0%) | 3 (4%) | p=0.468 |
| Normal weight (n, %) | 7 (27%) | 27 (33%) |  |
| Overweight (n, %) | 14 (54%) | 32 (39%) |  |
| Obese (n, %) | 5 (19%) | 21 (25%) |  |
